# Metabolic differentiation of surface and invasive cells of yeast colony biofilms revealed by gene expression profiling

**DOI:** 10.1186/s12864-017-4214-4

**Published:** 2017-10-23

**Authors:** Jana Maršíková, Derek Wilkinson, Otakar Hlaváček, Gregor D. Gilfillan, Alexandru Mizeranschi, Timothy Hughes, Markéta Begany, Stanislava Rešetárová, Libuše Váchová, Zdena Palková

**Affiliations:** 1 Department of Genetics and Microbiology, Faculty of Science, Charles University, BIOCEV, 252 50 Vestec, Czech Republic; 2 Institute of Microbiology of the Czech Academy of Sciences, BIOCEV, 252 50 Vestec, Czech Republic; 3 Oslo University Hospital and University of Oslo, 0450 Oslo, Norway; 4 NORMENT, Institute of Clinical Medicine, University of Oslo, 0450 Oslo, Norway

**Keywords:** *Saccharomyces cerevisiae*, Colony biofilms, Cell differentiation, Invasive cell subpopulation, Transcriptomics, Regulation of glycogen metabolism

## Abstract

**Background:**

Yeast infections are often connected with formation of biofilms that are extremely difficult to eradicate. An excellent model system for deciphering multifactorial determinants of yeast biofilm development is the colony biofilm, composed of surface (“aerial”) and invasive (“root”) cells. While surface cells have been partially analyzed before, we know little about invasive root cells. In particular, information on the metabolic, chemical and morphogenetic properties of invasive versus surface cells is lacking. In this study, we used a new strategy to isolate invasive cells from agar and extracellular matrix, and employed it to perform genome wide expression profiling and biochemical analyses of surface and invasive cells.

**Results:**

RNA sequencing revealed expression differences in 1245 genes with high statistical significance, indicating large genetically regulated metabolic differences between surface and invasive cells. Functional annotation analyses implicated genes involved in stress defense, peroxisomal fatty acid β-oxidation, autophagy, protein degradation, storage compound metabolism and meiosis as being important in surface cells. In contrast, numerous genes with functions in nutrient transport and diverse synthetic metabolic reactions, including genes involved in ribosome biogenesis, biosynthesis and translation, were found to be important in invasive cells. Variation in gene expression correlated significantly with cell-type specific processes such as autophagy and storage compound accumulation as identified by microscopic and biochemical analyses. Expression profiling also provided indications of cell-specific regulations. Subsequent knockout strain analyses identified Gip2p, a regulatory subunit of type 1 protein phosphatase Glc7p, to be essential for glycogen accumulation in surface cells.

**Conclusions:**

This is the first study reporting genome wide differences between surface and invasive cells of yeast colony biofilms. New findings show that surface and invasive cells display very different physiology, adapting to different conditions in different colony areas and contributing to development and survival of the colony biofilm as a whole. Notably, surface and invasive cells of colony biofilms differ significantly from upper and lower cells of smooth colonies adapted to plentiful laboratory conditions.

**Electronic supplementary material:**

The online version of this article (doi:10.1186/s12864-017-4214-4) contains supplementary material, which is available to authorized users.

## Background

Yeast biofilms and colonies are dynamic heterogeneous communities of differentiated yeast cells that exhibit specific spatio-temporal organization. The properties, fitness and functions of differentiated cells, on one hand, depend upon the precise environmental conditions at a cell’s location within the colony structure. On the other hand, the genetic/epigenetic traits of a yeast strain and the availability of nutrients help to shape the characteristics of the environmental niche during colony development. *Saccharomyces cerevisiae* strains adapted to high nutrient levels (particularly to high glucose) form smooth colonies of typical ovoid yeast cells that are tightly packed, with the colony growing on and above the surface of semisolid material such as agar. Wild *S. cerevisiae* strains (like other yeast, including pathogens) must cope with the stressful conditions of the natural environment and form colony biofilms with complex characteristics [1, 2]. The cells of these strains often perform a dimorphic switch, resulting in the formation of pseudohyphae that invade semi-solid surfaces. These cells produce extracellular matrix (ECM), which contributes to structuring the internal colony environment. Colony biofilms growing on agar medium are composed of an surface “aerial” part, of ovoid cells localized above the agar, and agar-invasive part, that primarily consists of pseudohyphae forming the “roots” that attach the colony to the substratum. Several functionally specialized cell types are formed from the early stages of biofilm formation and further develop and cooperate during colony growth. Some of these cells evolve protective mechanisms that appear to participate in the environmental resistance of colony biofilms. These mechanisms include expression of active multidrug resistance pumps, typically present in surface cell layers over the colony, and production of ECM in the colony interior [3]. Eventually, wild strains domesticate and begin to form smooth colonies similar to colonies of laboratory strains [4]. Different factors and processes such as adhesin Flo11p, ECM and changes in chromosomal copy number have been associated with this switch [5–8].

Genome-wide studies of cell subpopulations that were separated from smooth colonies of laboratory *S. cerevisiae* strains by gradient centrifugation or micromanipulation, revealed key information regarding the complexity of smooth colonies and the properties of, and mutual interactions among, the differentiated cells [9, 10]. However, similar information on colony biofilms is still missing, mostly because of the challenges of isolating invasive cells embedded in agar and ECM and of subpopulation cross-contamination. A few transcriptomic analyses have been limited to cells from surface parts [4, 7, 11]. To fill this gap in our knowledge, we have used a new method to isolate cells from colony invasive parts for extraction of RNA of sufficient quality for high-throughput RNA sequencing (RNA-seq). Genome wide transcriptional profiling revealed complex differences between surface (“aerial”) and invasive (“root”) cells with high sensitivity and reproducibility. These data, supplemented by assessing of functionality of identified processes and positioning of cells producing selected proteins within the colony structure allowed us to propose a model of the metabolic pathways that operate in root and aerial cells. Furthermore, we identified a major role for the Gip2p regulatory subunit of Glc7p phosphatase in glycogen accumulation in aerial cells. Comparison of novel data with previously identified characteristics of cells forming smooth, less structured colonies [9] shed new light on different lifestyles of yeast multicellular populations.

## Results

### Root and aerial cell separation and genome-wide transcription profiling

**Fig. 1:**
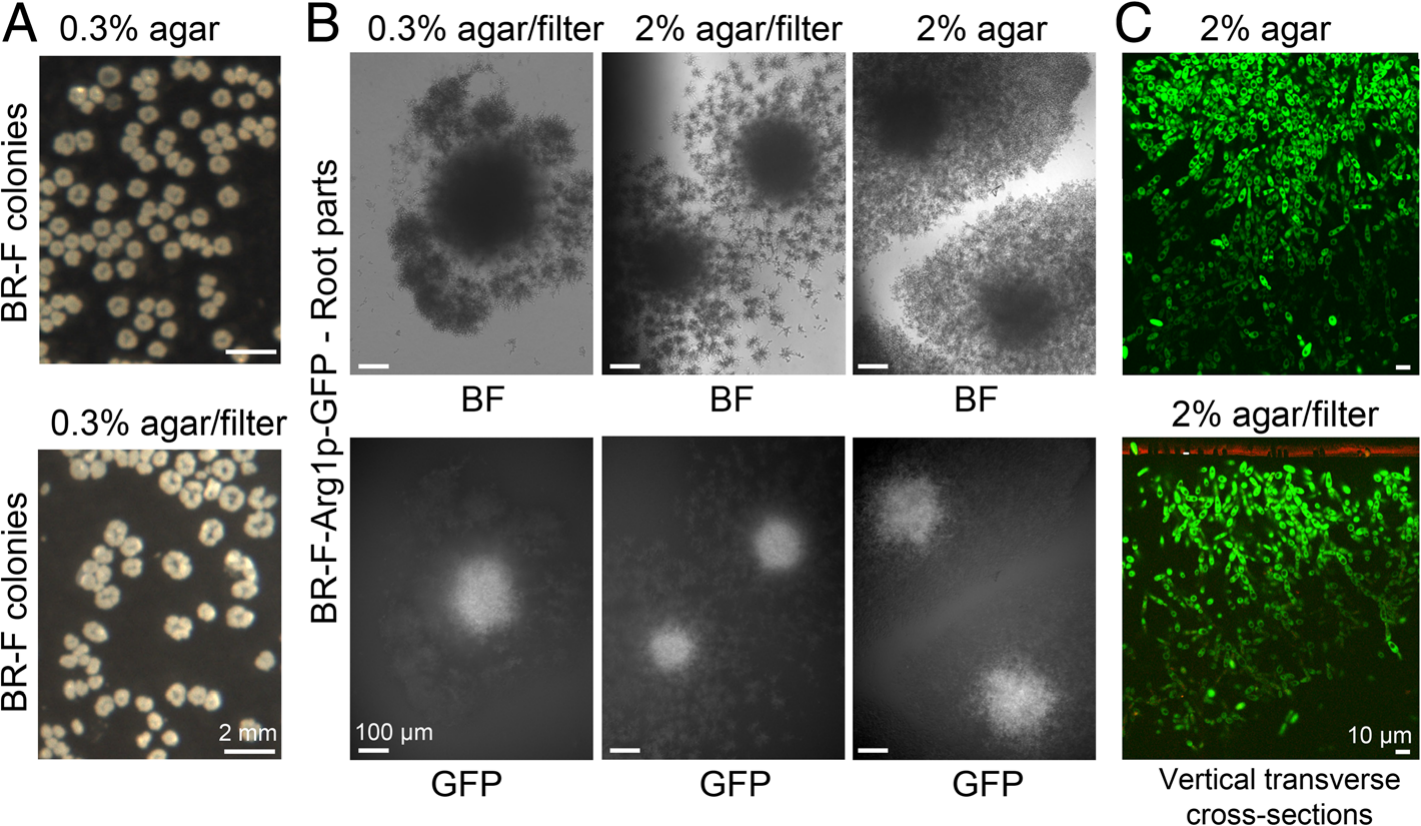
Morphology of colony biofilms cultivated on filters. **a** 3-day-old BR-F colonies cultivated on GMA medium with 0.3% agar in the absence or presence of a filter. **b** Aerial view of root parts of colony biofilms of the BR-F-Arg1p-GFP strain cultivated as in (**a**). BF, bright field; GFP, GFP fluorescence. **c** Side view of a vertical cross-section of root parts of colony biofilms of the BR-F-Arg1p-GFP strain cultivated as in (**a**) using 2PE-CM. Green, GFP fluorescence, red, filter autofluorescence

To elucidate the overall metabolic differences between aerial and root cells of colony biofilms formed by diploid wild strain BR-F, we used genome-wide transcription profiling. First, we developed a new technique to separate root (subsurface) cells from the agar and achieve sufficient purity to allow for efficient total RNA extraction (see Methods). We took advantage of the fact that colony biofilms growing on filters of certain porosity form typical morphology, including characteristic roots that invade through the filter into the agar, and produce similar levels of marker proteins to those in colony biofilms grown without filters (Fig. 1). This filter setup facilitates further transcriptomic analyses because it avoids cross-contamination of cell samples.

**Fig. 2:**
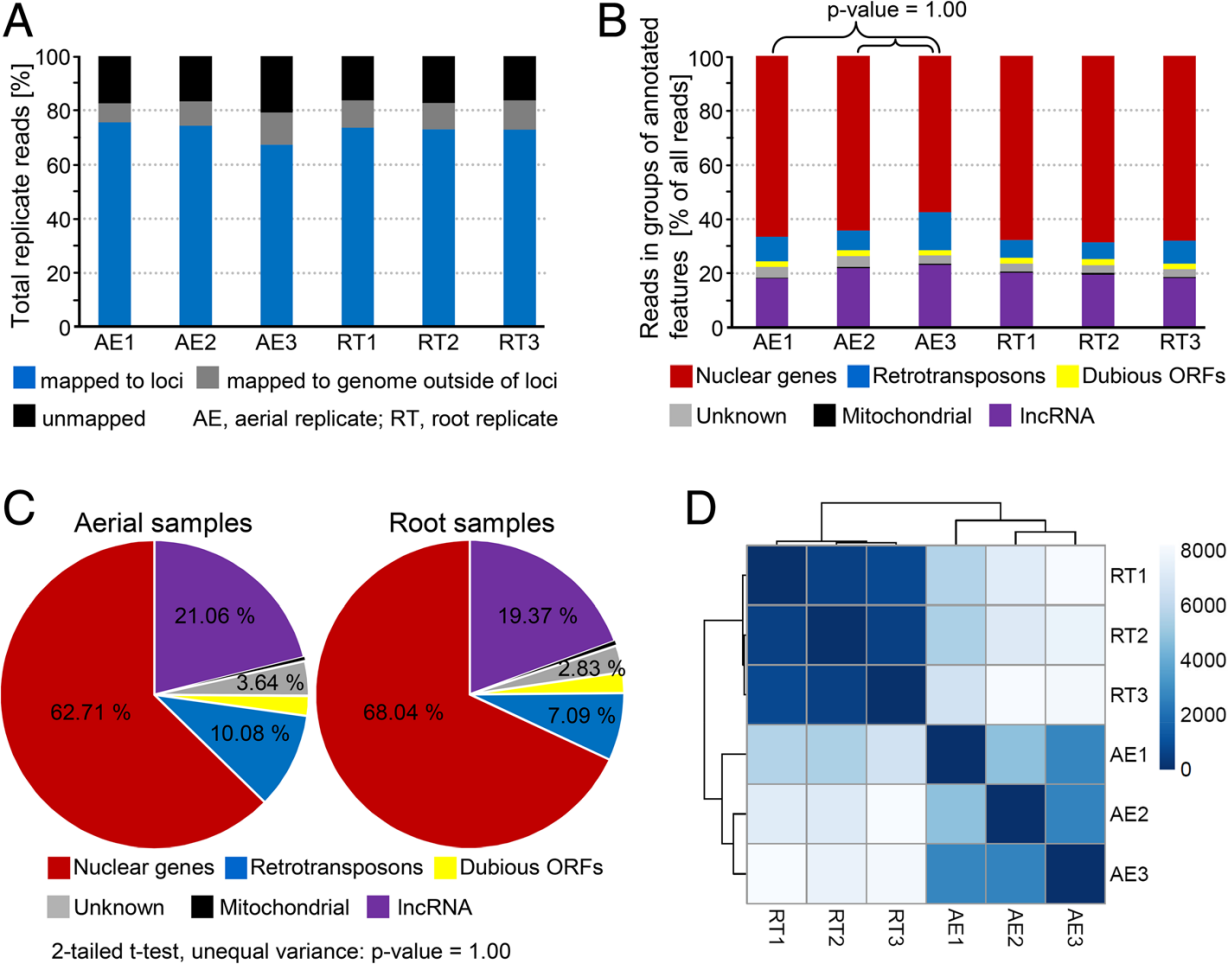
Read alignment statistics and counting results for individual replicates of aerial (AE) and root (RT) samples. **a** Percentages of reads mapping to annotated gene loci or intergenic regions and unmapped reads. **b** Percentages of reads mapped to lncRNA loci, mitochondrial loci, retrotransposons, loci encoding putative/unknown proteins, dubious ORFs and other nuclear loci. **c** Mean percentages of aerial and root sample reads mapped to different feature types. **d** Sample similarity map generated in R using the Bioconductor package; intensity of color is proportional to similarity, measured by Poisson distance

For the transcriptomic profiling described below, aerial and root cells were separated from 3-day-old colonies grown on filters. The RNA was extracted from three independent biological replicates of aerial and root samples. Each of the cell samples was harvested from at least 3000 colonies. The cDNA was prepared and sequenced using the Illumina HiSeq sequencing platform with strand-specific library preparations, yielding a high-quality, high-coverage transcriptome library with ~13.7-17 million sequencing reads per sample mapped to 10,240 loci of coding and noncoding RNAs or to intergenic regions of the yeast genome. For all replicates, more than 79% of reads mapped to the yeast genome (Fig. 2a), more than 57% of mapped reads aligned to nuclear genes, and 23% were mapped to long non-coding RNA (lncRNA) loci (Fig. 2b). Visual inspection, using the Integrative Genomic Viewer [12], of many randomly selected sites with genes on both strands confirmed that the strand information had been retained. Although a slightly lower percentage of aerial reads mapped to genes and slightly higher percentages mapped to lncRNAs and to retrotransposons (Fig. 2c), a 2-tailed Student’s t-test with unequal variance comparing the percentages of genes matching to different feature types produced a *p*-value of 1.0, indicating that the two cell types are not different in gross expression. Aerial replicate 3 appeared to be slightly different than the other two aerial replicates in terms of read mapping to different types of loci (Fig. 2a, b, d), but Student’s t-tests comparing replicate 3 to replicate 1 and replicate 2 returned a p-value of 1.0, indicating that the distributions are essentially similar. Plots of the log ratios of expression levels for all genes in a chromosome (Additional file 1: Figure S1) show no extensive areas of generally upregulated or downregulated expression that would indicate the occurrence of gene duplication/loss in aerial or root cells.

On average, 62.7% of mapped aerial reads and 68.0% of mapped root reads (Fig. 2c) mapped to nuclear loci encoding characterized proteins and 10.1, 21.1 and 3.6% of aerial reads and 7.1, 19.4 and 2.8% of root reads mapped to retrotransposons, loci encoding lncRNAs and nuclear loci encoding putative proteins, respectively. The remainder (< 2.7% in each case) mapped to mitochondrial loci or to dubious ORFs.

Differential expression results were generated for 10,218 loci (Additional file 2: Table S1). Small fold changes between cell types (even if statistically significant) are of limited interest, so we focused on genes with expression fold changes of two or greater in the differential expression results. We found 1245 differentially expressed protein-coding genes with a *p*-value of 5% or less. Of these, 1197 retain significance when adjusting for multiple testing with a false discovery rate (FDR) of 5% and 714 genes remain when applying the most stringent Bonferroni correction (Additional file 2: Tables S2 and S3). The equivalent results for lncRNA are 420 (p-value <5%), 229 (adjusted *p*-value less than 5% FDR), and 68 (Bonferroni correction: p-value <4.8E-06) [13].

**Fig. 3:**
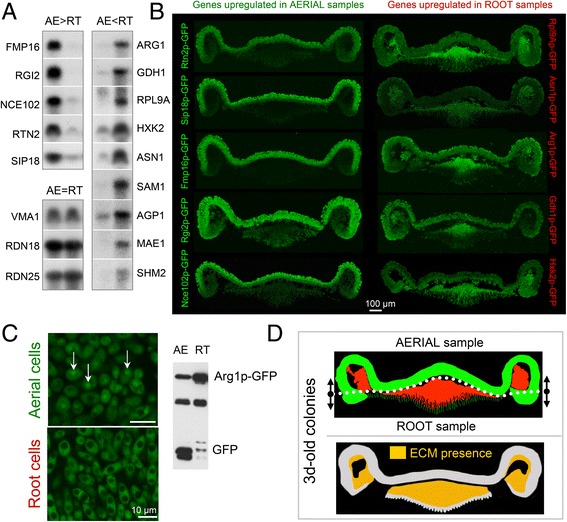
Analyses of marker genes and proteins and functions of selected processes in aerial and root cells. **a** Northern blot analysis of selected RNAs with higher expression in aerial (AE) and in root (RT) samples, identified by RNA seq. *VMA1*, *RDN18* and *RDN25* are unregulated controls. **b** Levels of GFP-labeled proteins (expected to be more highly expressed in aerial or root samples based on the expression data) in 3-day-old colonies of respective BR-F derived strains (Additional file 2: Table S7). Vertical cross-sections of colonies were analyzed by 2PE-CM. Green, GFP fluorescence. **c** 2PE-CM of aerial and root cells of 3-day-old BR-F-Arg1p-GFP colonies (left). Western blot (WB) of Arg1p-GFP from aerial (AE) and root (RT) cells (right). In aerial cells, autophagy is active and cytosolic Arg1p-GFP is delivered to vacuoles (indicated by white arrows) (left) and degraded as indicated by free GFP in WB (right). **d** Model of localization of cells with aerial features (in green) and root features (in red) in 3-day-old colony biofilms based on the levels of GFP-labeled proteins in different colony areas as shown in B. Dotted white line indicates border between cells collected as aerial cells and root cells (upper panel). One feature of this model is a spatial overlap between cells with root features and cells embedded in ECM and between cells with aerial features and ECM-free cells, as described in [3]; ECM is indicated by yellow color in the lower panel of the scheme (reprinted from [3])

The unusually large number of genes that remain significantly differentially expressed after correction for multiple testing is the result of the substantial transcriptomic differences between these cell types and of our experimental procedures and design. The cell separation method with three replicates for each cell type and very deep sequencing of the samples likely significantly contributed to the robustness of our results to correction for multiple testing. The aerial-root differences in expression (Additional file 2: Table S1) of selected genes were confirmed by northern blot hybridization (Fig. 3a) and production of selected proteins by GFP tagging and microscopy (Fig. 3b).

**Fig. 4:**
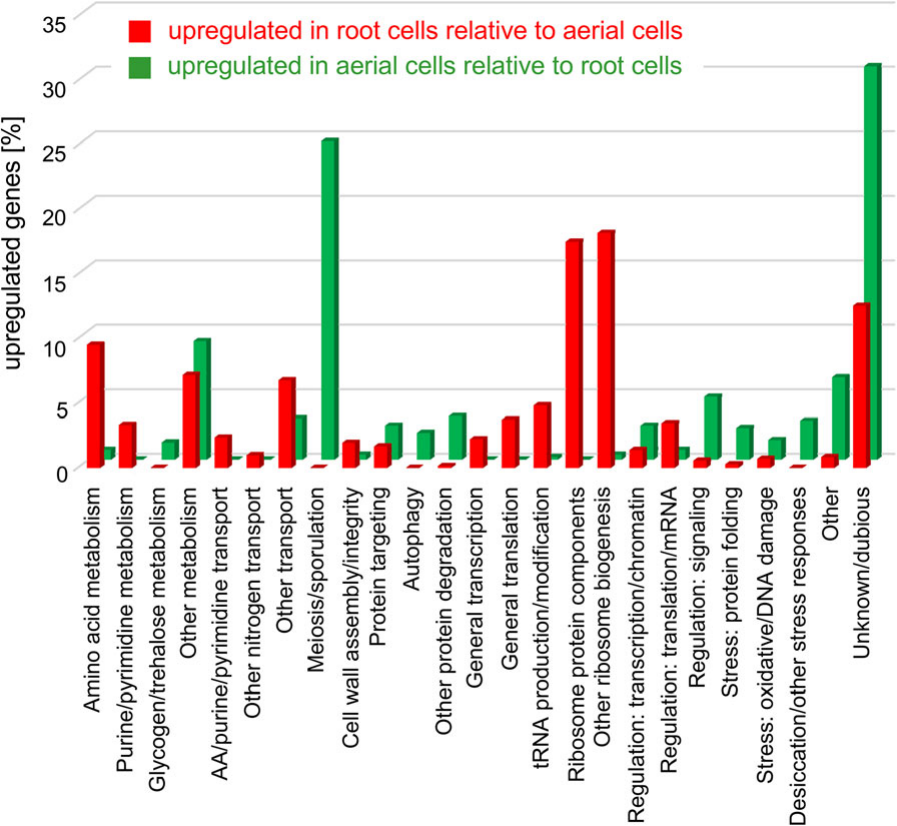
Functional gene groups, upregulated in aerial and in root cells. Following manual annotation, coding genes that were upregulated (at least two-fold and with a *p*-value <0.05) in root or in aerial cells were clustered according to functional group and the percentages of upregulated genes in each group were calculated

Next, we performed global functional characterization of the differentially expressed genes using Gene Ontology (GO) analysis and hierarchical clustering, further controlled and modified by manual assessment of individual gene functions based upon information in the SGD (http://www.yeastgenome.org/) and the literature. This complex analysis revealed different biological processes to be upregulated in aerial or root subpopulations as compared with the opposite subpopulation, i.e., upregulated in aerial versus root and vice versa (Table 1, Fig. 4). It is worth noting here that the term “upregulation” of gene expression used throughout the text is always relative to the opposite subpopulation and does not imply that a gene-expression difference is due to an increased rate of transcription in one subpopulation or decreased rate in the other. In general, root-upregulated genes are enriched for genes involved in metabolic processes. The major gene groups upregulated at least two-fold almost exclusively in roots are involved in ribosome biogenesis or structure (259 genes), translation (62 genes), amino acid (69 genes) and purine/pyrimidine (24 genes) biosynthesis. In contrast, the largest groups of genes upregulated exclusively in aerial parts include genes involved in meiosis and sporulation (134 genes), in stress responses (37 genes) and in protein degradation (29 genes, mainly autophagy and the ubiquitin-proteasome system). These differences and other differences in gene expression, discussed below, indicate that cells of the root subpopulation activate numerous biosynthetic processes and aerial cells decrease synthetic processes and reprogram their metabolism to cope with starvation and different stresses.

**Table 1:** Functional grouping of differentially expressed genes in aerial and root cells

	Genes more highly expressed in aerial cells	Genes more highly expressed in roots
Metabolism		
Amino acid metabolism	CAR1, GAD1, ARO9, ARO10	69 genes
Purine, pyrimidine synthesis		24 genes
Glycogen/trehalose	GLG2, GDB1, GPH1/TPS1, TPS2, TSL1, IGD1	
Nitrogen metabolism - other/Glycolysis, gluconeogenesis/Pentose phosphate shunt/ TCA, glyoxylate cycle/Carbohydrate metabolism - other/ Respiration, OxPhos/Cofactor, vitamins/Lipid metabolism	FBP26, ENO1, HXK1, PGK1, TDH1/GND2, NQM1, PRM15, SOL4, TKL2, YEF1 / IDP3, YPL113C/ALD2, ALD3, AMS1, FDH1, GAL7, GRE3, NCA2, SYM1, XKS1, XYL2, YDL124W, YIG1, YJR096W/COQ4, COA4, SHH4, RCF1, MCR1, NDE2/PCD1, THI4, THI11, YNL200C/AYR1, CTA1, ETR1, HES1, HMG2, HFD1, OPI10, SRT1, TGL1, FAA2, FOX2, POX2, POT1	DUR1,2, DAL1, DAL2, DAL3/FBP1, HXK2, PFK27/ TKL1, RKI1, SHB17/ACO2, CIT2, ICL1, MAE1, MLS1/ACS2, ACH1, ADH1, ADH3, ADH5, ADH6, ALD5, ALD6, DLD2, DLD3, GPI18, IMA5, MNN1, MTD1, PCS60/COQ2, CYC1, HEM1, HEM13, OYE2, Q0085, RCF2 YAH1/BUD16, BUD17, HEM3, ISU2, PAN6, PHO3, SPE4/CDS1, ERG3, ERG4, OLE1, SCS3, SUR2, SUR4
Transport		
Amino acids / purine, pyrimidine		AGP1, BAP2, BAP3, CAN1, DIP5, GAP1, HIP1, PUT4, SAM3, TAT1, TAT2, YMC1, YMC2 / FCY2, FCY22, FUI1, FUR4
Nitrogen - other		ATO2, DAL4, DUR3, MEP1, MEP2, MEP3, TPO1
Other transport: Sugars/Carbohydrates - other/Other ions/Others	GAL2, HXT5, HXT6, HXT14, YDL199C, YFL040W, VPS73/ − / CTR3, PMA2, MFM1, ADP1, PRM6/BPT1, PDR10, PDR18, PXA1, PXA2	HXT1, HXT2, HXT4, HXT13, HXT17/CRC1, CTP1, MPC2, JEN1, OAC1, ODC2, SFC1, VTC1, YAT1, YAT2, YHM2 / AST1, ENB1, FET3, FSF1, FTR1, GDT1, IZH4, MIR1, MMT1, PHO84, PHO88, PHO89, PMA1, PMP2, SIT1, ZRT1, ZRT2 / ANT1, AQR1, AQY2, ATR1, GDA1, GGC1, HNM1, OPT2, PET9, PRY2, PDR5, PTR2, RSB1, THI7, TNA1, VRG4
Meiosis, sporulation	134 genes	
Cell wall (assembly, integrity)	DAN4, YGP1	CIS3, CRH1, FIT2, FKS1, GAS1, HSP150, KTR2, PUN1, SCW10, TOS1, TOS6, UTH1, UTR2, YPS3,
Protein targeting		
Endocytosis, vesicle sorting/Mitochondria/Nucleus/Peroxisome	BTN2, COS4, EMP46, ERV29, GGA1, LSP1, ROD1, SIP3, SNX41, ERP3 / − / POM33/ PEX18, PEX22, PEX1	ART5, COS10, EMP70, MRS6, RTT10, YIP1/OXA1, PAM18/ BCP1, KAP123, NMD5, PSE1
Protein degradation		
Autophagy/ Ubiquitin, proteasome/ Other	ATG2, ATG3, ATG7, ATG8, ATG9, ATG14, ATG16, ATG17, ATG34, ATG39, YOR152C/ GID COMPLEX: FYV10, VID22, VID28, VID30, RMD5; ASI1, CUZ1, ECM29, HUL4, RRI2, SAF1, UBC11, UBP2, UBR2, YUH1/ PAI3, PRD1, YBR139W	−/RFU1/−
General transcription		16 genes
General translation/tRNA production, Modification/Ribosome proteins/ Biogenesis	−/SOL1/−/RNP1, SRD1	27 genes/35 genes/127 genes/132 genes
Regulation		
Transcription/Chromatin remodeling/ Translation, mRNA export, degradation/ Signaling	AZF1, CRF1 GCR1, MSA2, NRM1, XBP1 / ASF2, EPL1, ESC8, HST4, RIF1, RTT109, SPT21, SWI1/ HEF3, STO1, NGR1, MIP6 / AXL1, BEM4, BMH2, CYR1, FAR1, GIP2, HO, HYM1, KSP1, NNK1, PBP2, PHO92, PIG1, PIG2, PKH2, PRR2, RAD53, STE3, STE18, TEL1, TFS1, TPK1, TPK2, VHS3, VPS15	ARG80, CAF16, NRG1, SUT1, SUT2, VHR2 / FPR4, NPT1, SMP1, TOD6/ YIH1, ASC1, ANB1, DPH1, JJJ3, DPH2, ADF1, CAF20, CDC33, DPH5, DYS1, EFM1, EFM2, EFM4, GCD7, GCN3, LIA1, PUF6, RBG1, RRT2, SUP45, TEF1, TIF1, TIF5, TMA19/ PPT1, SPL2, INM1, YVH1
Stress response		
Protein folding/Oxidative stress/Dessication/Other stress	ECM10, HSC82, HSP104, HSP26, HSP42, HSP78, HSP82, SSA1, SSA3, SSA4, SSE2, STI1, TMA17/CTT1, GPX1, GTO1, TRR2, RNY1/GRE1, SIP18, STF2/DCS2, DDR2, ECM4, GLO1, GTO3, HSP33, SED1, SNO4, SPG1, SPG4, YJL144W, YKL151C, YRO2/MSH2, PHR1, ULP2	EMC6, PAC10/ CCS1, FRM2, GPX2, SRX1, TRR1/−/−
Others	34 genes	6 genes
Unknown/dubious	158 genes	85 genes

### Specific pattern of aerial and root cell proteins in colony biofilms

Based on the transcriptomic profiling, we selected genes that were more highly expressed in either aerial or root cells and analyzed the levels of their protein products labeled with GFP in colony biofilms using colony cross-sectioning and two-photon excitation confocal microscopy (2PE-CM) (Fig. 3b). In agreement with the expression data, the levels of most of the GFP-labeled proteins differed significantly in aerial and root parts. However, the differences between aerial and root parts for proteins encoded by aerial-upregulated genes were generally higher than predicted by the transcriptomic data whereas differences in root-marker genes were often lower than predicted. The aerial:root protein ratios were roughly estimated based on fluorescence microscopy because our attempt to prepare root samples suitable for reproducible protein quantification was unsuccessful due to traces of medium in the samples (not shown). Northern blot hybridization confirmed expression data (Fig. 3a), so differences in protein levels indicate reduced efficiency of mRNA translation or increased protein degradation in roots than in aerial cells.

2PE-CM analysis confirmed specific complementary localization patterns of cells expressing proteins encoded by “aerial” genes and those encoded by “root” genes (Fig. 3d) and revealed further details of localized expression. As expected, cells producing “aerial” proteins localize predominantly to aerial parts of colonies but most of the “aerial” proteins are also abundant in a tiny cell fraction at the tips of the roots that invade the agar but not in cells within the cavities of aerial parts. In contrast, cells expressing abundant “root” proteins localize mainly to the upper part of the root area and to cavities of aerial parts. In addition, most of these proteins are not present (or are very scarce) in cells at the tips of the roots.

### Biosynthetic processes and transport of metabolites in root cells

**Fig. 5:**
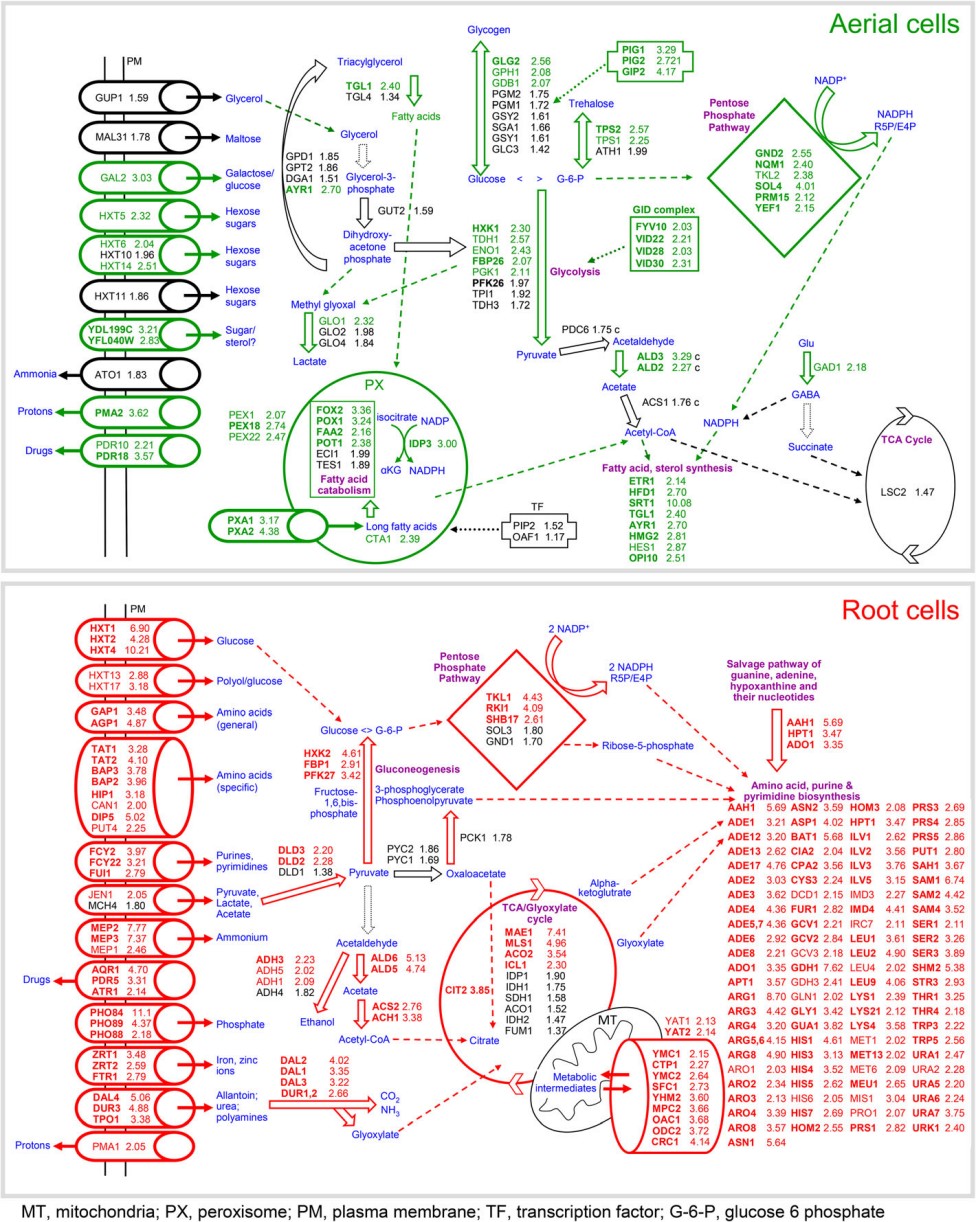
Model of metabolic processes upregulated in aerial and in root cells. Gene and metabolic pathway information from Yeast Pathways (http://pathway.yeastgenome.org/), from SGD (http://www.yeastgenome.org/) and the literature was used to identify upregulated metabolic pathways and groups of transporters in both cell subpopulations and construct model schemes representing metabolic differences between aerial and root cells. Differentially expressed genes that were found to be significantly differentially expressed even after application of the Bonferroni correction for multiple testing are shown in bold

Upregulation of a large group of genes involved in amino acid metabolism and genes encoding different amino acid transporters is a prominent characteristic of root cells (Fig. 5, Table 1). The transporters differ in specificity, capacity, and thus in ability to import specific amino acids present at varying quantities in the growth medium, from high concentrations to traces. To distinguish whether root cells accumulate free amino acids or utilize them for protein biosynthesis, we extracted amino acids from aerial and root cells of 3-day-old colonies and quantified the amino acids using HPLC. The results (Additional file 1: Figure S2) showed that intracellular amounts of free amino acids are similar in aerial and root cell subpopulations. This finding supports the second alternative, which is further supported by the higher expression of a large group of genes involved in ribosome biogenesis and other ribosomal functions specifically in roots (Table 1). Thus, the data indicate that both synthesized and imported amino acids are rapidly used for protein biosynthesis and biomass increase in roots.

The metabolic genes upregulated in roots include genes with roles in specific branches of carbohydrate metabolism (Fig. 5). There are several indications that gluconeogenesis is upregulated in root cells: *FBP1*, encoding the major gluconeogenic enzyme fructose-1,6-bisphosphatase, is upregulated more than 2.9 fold, and three other gluconeogenesis genes, *PYC1* and *PYC2* for pyruvate carboxylase and *PCK1* for phosphoenolpyruvate carboxykinase, more than 1.6 fold. Interestingly, *HXK2*, encoding the hexokinase isoenzyme (responsible for glucose phosphorylation, the first step of glycolysis) that is the predominant hexokinase during growth in glucose medium [14], is also highly upregulated in root cells (4.6-fold). This finding suggests that any glucose entering the cells would be efficiently converted to glucose-6-phosphate, i.e., to a form that stays within the cells and is utilized in different metabolic pathways, including the pentose phosphate pathway (PPP) that yields pentoses, essential to the biosynthesis of some amino acids, nucleosides and nucleic acids. The gluconeogenesis/glycolysis intermediate 3-phosphoglycerate can enter serine biosynthesis, the first step of which is mediated by 3-phosphoglycerate dehydrogenase (*SER3*, ~4-fold upregulated), and subsequently glycine metabolism and one-carbon metabolism needed for methionine and purine biosynthesis.

Increased tricarboxylic acid (TCA) cycle and/or glyoxylate cycle activities in root cells are indicated by upregulation of the *ACO2* gene for aconitase (3.5-fold), *ICL1* for isocitrate lyase (2.3 fold) and *CIT2* for a citrate synthase isoform (3.9-fold) and by upregulation of a group of genes encoding carriers shuttling different metabolite intermediates between mitochondria and other cellular compartments (11 genes, over 2-fold upregulation). The glyoxylate cycle is also believed to be essential for the gluconeogenic mode of metabolism [15] that is characteristic of root cells. In addition, *ALD5* and *ALD6*, which encode NADP^+^-specific aldehyde dehydrogenases, are highly upregulated (approximately 5-fold) and, with *ACS2* acetyl-coA synthetase (2.8-fold upregulated), could contribute to acetyl-CoA production as well as to the maintenance of redox-state in root cells. Allantoin/urea transporters and catabolism (>2.5-fold upregulation) could provide additional intermediates such as glyoxylate.

### Storage compounds and metabolism in aerial cells

**Fig. 6:**
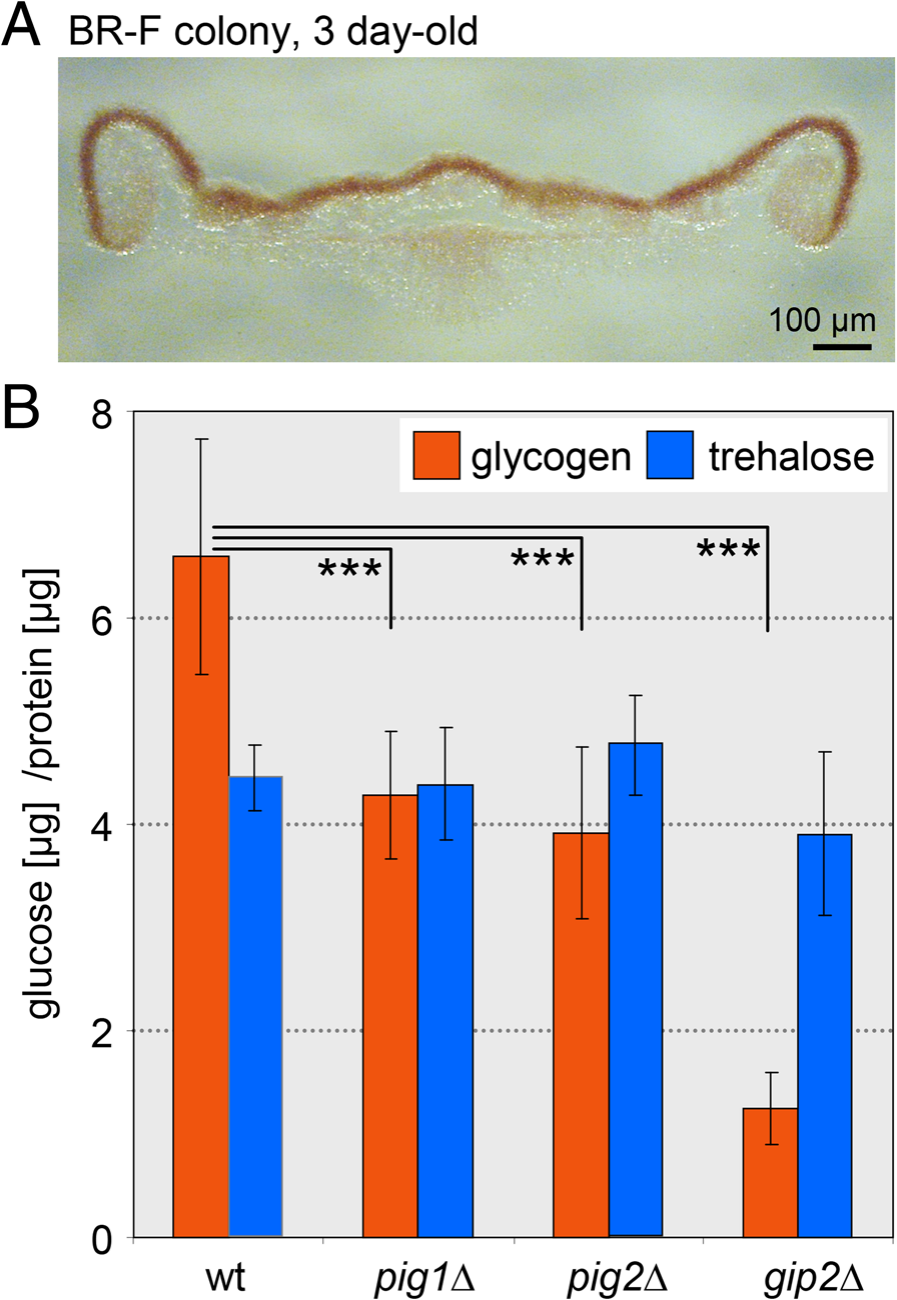
Glycogen and trehalose content in colonies. **a** Presence of glycogen (in brown) in 3-day-old BR-F colonies. Thin cross-sections of colonies were stained and observed using transmitted light. **b** Glycogen and trehalose content in aerial cells from wt, *gip2*Δ, *pig1*Δ and *pig2*Δ colonies. The mean of three biological and 3-4 technical replicates each is shown ± SD, ***p < 0.0001

Both biosynthetic and catabolic genes for proteins involved in glycogen and trehalose metabolism are upregulated in aerial cells (Fig. 5), indicating increased turnover of these storage polymers and glucose. Staining of glycogen in cross-sections of 3-day-old BR-F colonies by iodine vapor showed a significantly higher level of glycogen in aerial than root cells (Fig. 6a), demonstrating that synthesis of glycogen prevails over its degradation in aerial cells. Glycogen synthesis can be regulated via modulation of activity of Gsy2p glycogen synthase [16]. *PIG2*, *PIG1* and *GIP2* genes for putative regulatory subunits of Glc7p phosphatase involved in dephosphorylation and activation of Gsy2p were among genes highly upregulated in aerial cells (Fig. 5). We therefore examined the potential role of these genes in glycogen accumulation in colony biofilms of knockout strains. Deletion of any of these genes diminished level of glycogen in aerial cell whereas the level of trehalose remained almost unaffected (Fig. 6b). *GIP2* deletion exhibited the most prominent effect, whereas deletions in *PIG1* and *PIG2* were rather moderate.

Several catabolic pathways are upregulated in aerial cells (Fig. 5, Table 1). Upregulated peroxisomal functions in aerial cells are indicated by expression of genes for peroxisome biogenesis (*PEX1*, *PEX22* and *PEX18*), transport of fatty acids (*PXA1* and *PXA2*) and fatty acid β-oxidation (*FOX2*, *POX1*, *FAA2, POT1* and *IDP3*) leading to the release of acetyl CoA. Upregulation of *ALD3* and *ALD2* aldehyde dehydrogenases and *ACS1* acetyl CoA synthetase is another sign of acetyl CoA production in aerial cells. Acetyl CoA could be utilized in the TCA/glyoxylate cycle or for fatty acid and sterol synthesis. Several genes associated with lipid metabolism are upregulated at least two-fold in aerial cells, including *HES1*, *HMG2* and *TGL1*, which are involved in sterol biosynthesis, and *ETR1*, *HFD1*, *OPI10*, *SRT1* and *AYR1*, with roles in fatty acid, sphingolipid, phospholipid, dolichol (used in N-glycosylation) and phosphatidic acid biosynthesis. *SRT1* is upregulated 10-fold, suggesting that protein glycosylation may be an important process in aerial cells.

There are indications that glycolysis is active in aerial cells, and some genes with dual roles in both glycolysis and gluconeogenesis (such as *TDH1* and *ENO1*) are upregulated in aerial cells. Also upregulated in aerial cells is a group of genes coding for proteins of the GID complex, which has ubiquitin ligase activity and is implicated in the degradation of fructose-1,6-bisphosphatase and phosphoenolpyruvate carboxykinase (gluconeogenic specific enzymes) and thus in the inhibition of gluconeogenesis during cell transition from gluconeogenesis to glycolysis (e.g., following a transfer from non-fermentable carbon sources to glucose). In addition, *GLO1*, encoding a glyoxalase that catalyzes the detoxification of methylglyoxal (a non-enzymatic side-product of glycolysis) via condensation with glutathione to yield S-D-lactoylglutathione, and *GLO2* and *GLO4,* which convert this intermediate to D lactate and glutathione, are also upregulated (2-fold and 1.8-fold respectively).

### Stress responses, the pentose phosphate pathway and redox states

Thirty-seven genes with roles in various stress responses were upregulated in aerial cells, compared to only seven genes found upregulated in roots (Table 1). Thirteen of these stress-response related genes in aerial cells encoded proteins involved in protein folding or refolding. Another four genes (*GRE1*, *SIP18, STF2* and *YJL144W*) are involved in desiccation-rehydration processes, three genes (*MSH2*, *PHR1* and *ULP2*) in DNA repair and five genes in oxidative stress defense (*CTT1*, *GTO1*, *TRR2*, *RNY1* and *GPX1*). Five of the seven genes upregulated in root cells are involved in oxidative stress response (*GPX2*, *TRR1*, *SRX1*, *FRM2* and *CCS1*).

Genes for alternative isozymes of transketolase, transaldolase, 6-phosphogluconolactonase and 6-phosphogluconate dehydrogenase involved in PPP were upregulated in aerial (*TKL2*, *NQM1*, *SOL4* and *GND2*) and root (*TKL1*, *SOL3* and *GND1*, the last two genes 1.8-fold and 1.7-fold respectively) cells (Fig. 5), indicating that PPP output is shifted towards precursors of amino acid/nucleotide biosynthesis in roots and towards production of NADPH (and thus redox-state balancing) in aerial cells [17]. The *RKI1* gene for ribose-5-phosphate ketol-isomerase, which generates important precursors of amino acid biosynthesis from the pentose phosphate pathway [18], was also upregulated in roots. This finding fits with the observation of upregulated amino acid metabolism in roots and increased stress responses of cells in aerial regions.

### Autophagy in aerial cells

Autophagy genes (11 genes) are upregulated in aerial cells compared with root cells. 2PE-CM of cross-sections of colonies of BR-F producing cytosolic or peroxisomal GFP-tagged proteins showed accumulation of GFP in vacuoles of aerial cells (a sign of active autophagy delivering cytosolic proteins/peroxisomes to the vacuole for degradation) (Fig. 3c). No GFP was observed in the vacuoles of root cells. Active autophagy in aerial cells was confirmed by western blot of aerial/root cells of an Arg1p-GFP-producing strain; the tagged cytosolic protein is degraded to free GFP in the vacuoles only in aerial cells. This observation fits with the transcriptomics data and indicates that autophagy is active in aerial cells but not in the root cells.

### Expression of alternative isozymes indicates different glucose levels in root and aerial parts

As shown in Fig. 5, isogenes of different metabolic enzymes and some transporters are differently expressed in aerial and root samples. Isogenes that are more highly expressed in carbon-limited (low glucose) conditions, such as *GND2*, *TKL2*, *NQM1*, *ACS1*, *HXK1*, *PDC6*, *ALD2*, *ALD3*, *GAL2*, *HXT5*, *HXT6*, *HXT10*, and *HXT14*, are upregulated in aerial samples and those that are more highly expressed on fermentable carbon sources, such as *GND1*, *TKL1*, *SOL3*, *ACS2*, *HXK2*, *PDC1*, and *ACO2*, are upregulated in root samples. As colonies were grown from the outset on respiratory GMA agar without glucose, potential differences in intracellular glucose/sugar levels could be due to differences in cell subpopulation metabolisms. Some glucose and/or other sugars can originate from polysaccharides of the extracellular matrix synthesized from glycerol earlier in colony development (ECM starts to be produced within ~ 30 h old colonies; [3]). In addition, observed isogene expression differences are consistent with an upregulation of gluconeogenesis that provides glucose to root cells and an upregulation of glycolysis that degrades glucose in aerial cells.

### Secreted proteins, the cell wall and adhesins

The secretion of ECM is an important feature of colony biofilms, but little is known about this process. Forty-two yeast orthologues of 38 known *Candida albicans* genes encoding secreted proteins [19] were identified (Additional file 2: Table S4) using the *Candida* genome database (http://www.candidagenome.org/). Nine of these orthologues were upregulated at least 2-fold in roots (Additional file 2: Table S4). These included six genes implicated in cell wall organization: two chitin transglycosylases (*UTR2*, *CRH1*), a β-1,3-glucanosyltransferase (*GAS1*), a yapsin family aspartic protease *YPS3*, a glucanase-like protein (*SCW10*), and a GPI-anchored protein of unknown molecular function (*ECM33*). In aerial cells, secretory protein genes with known roles in spore formation were upregulated: three orthologues of *C. albicans* secreted protein genes (Additional file 2: Table S4) were upregulated at least 2-fold in aerial cells (*CTS2*, *CRR1*, *SPR1*) and the gene *GAT4* that encodes a transcription factor involved in spore wall assembly (upregulated 1.7-fold).

In addition to ECM, Flo11p adhesin is the most important protein identified as essential to the formation of biofilm architecture [8]. No significant differences in *FLO11* mRNA levels were identified in this study, suggesting Flo11p plays a role in the entire colony.

### Non-coding gene expression

Our database of annotated long non-coding RNAs (lncRNAs) contains 3694 loci of different types: stable unannotated transcripts (SUTs) [20–22] and unstable lncRNA transcripts consisting of meiotic unstable transcripts, (MUTs: [20]), XUTs (Xrn1p-dependent unstable transcripts; [23]) and classical Rrp6p-dependent cryptic unstable transcripts (CUTs: [21, 24]). A total of 3658 of these transcripts were covered by at least one read in our study (Additional file 2: Tables S5 and S6). Four hundred and 20 of these loci were identified as differentially expressed (*p* < 0.05, fold difference > 2). Ninety-seven unstable transcripts (7 MUTs, 35 XUTs and 55 CUTs) and 79 stable transcripts (SUTs) were upregulated in roots relative to aerial parts. One hundred fifty-two unstable transcripts (102 MUTs, 39 XUTs and 11 CUTs) and 92 stable transcripts (SUTs) were upregulated in aerial parts relative to roots (Additional file 2: Table S5). Four hundred and five of these 420 upregulated loci lie within 1.5 kB of one or more coding genes (Additional file 2: Table S6).

## Discussion

Whereas laboratory and domesticated yeast strains form smooth colonies of cells that do not invade the agar and can be separated relatively easily [9, 25], wild yeast strains form complex colony biofilms with features of natural biofilms. Cells within colony biofilms are connected by extracellular fibers, and the lower parts of colonies (“roots”) are formed by pseudohyphae. The cells in the interior (including most of the roots) are embedded in protective ECM [3]. The root parts of colonies that invade the agar are difficult to harvest and purify, and we have to date lacked information on gene expression and regulation in these cells. To fill this gap in our knowledge, we used a new method for separation of root and aerial cells from colony biofilms. This method solves two major problems - the separation of root and aerial cells with minimal cross-contamination and the rapid purification of root cells suitable for extraction of RNA for expression profiling. This method enabled us to identify differences in transcript levels in aerial and root samples with high sensitivity. Despite a higher upregulation threshold (two-fold versus 1.5-fold), we identified more than twice as many upregulated genes and ten times as many upregulated lncRNAs per subpopulation as the microarray-based studies of laboratory strain colonies by [9] and [26]. High-throughput RNA sequencing provides a greatly increased dynamic range for quantification of gene expression [27] and is superior to microarrays for the detection of transcripts expressed at low levels. The lower number of differentially expressed lncRNAs detected in the study by Traven et al. [26] (213 genes, 14 CUTs and 16 SUTs upregulated on the inside and 312 genes, 5 CUTs and 7 SUTs on the outside of the colony) is probably partly due to the use of a smaller dataset of lncRNA loci (only the SUTs and CUTs identified by Xu et al. were analyzed) than in the current study. This study did not identify shorter ncRNAs because the magnetic bead purification method used excludes smaller RNAs.

2PE-CM analysis of GFP-labeled aerial and root marker proteins that were selected based on differential gene expression revealed additional complexity in both cell populations (Fig. 3b, d). Cells with “root” properties comprise the majority of root parts and localize to the cavities of aerial parts, whereas “aerial” properties are typical of cells in aerial parts and at the tips of the roots (Fig. 3d). This pattern closely resembles the pattern of cells embedded/non-embedded in ECM [3]: cells with “root” properties tend to be those embedded in ECM, whereas cells with “aerial” properties tend to be localized to ECM-free areas. Differential RNA expression profiling thus contributed to the identification of properties of minute cell sub-populations that cannot be separated from colony biofilms.

The RNAseq data, validated by northern blot analyses of selected transcripts and by determination of the level and localization of selected proteins in colonies, allowed us to develop a model of metabolic processes active in root (ECM-embedded) and aerial (ECM-free) cells. Metabolic implications were further confirmed by additional assays (e.g., monitoring of autophagy and measurement of glycogen and amino acid levels). Root cells appear to synthesize amino acids and glucose-6-P and use these intermediates for building new cellular components including cell walls (cell wall assembly genes are upregulated) and ECM. High-level expression of ribosomal genes and genes involved in translation indicates high anabolic activity in root cells. As dividing cells localize to the bases of roots and to the insides of aerial cavities [3], metabolic intermediates could be utilized during the growth and formation of new cell generations. Interestingly, several metabolic genes that are upregulated in root cells have been shown to be upregulated in liquid culture cells grown on glucose and their isogenes, which are upregulated in aerial cells, have been shown to be activated during glucose starvation. This suggests that root cells within colonies grown from the beginning on respiratory glucose-free agar medium can accumulate glucose (or other sugars) with local concentrations that are sufficient to upregulate glucose-induced isogenes. Such glucose could be formed by gluconeogenesis (which is upregulated in root cells) or provided by aerial cells (for example, by degradation of extracellular trehalose by acid trehalase in the periplasmic space; the gene encoding acid trehalase *ATH1* is upregulated almost 2-fold in aerial cells). In addition, ECM retains a lot of water [28] and can function as a reservoir of water-soluble metabolites, including sugars. Moreover, ECM is composed primarily of polysaccharides, as indicated by Concanavalin staining [28], and sugars may be released following ECM hydrolysis under specific conditions. Various nutritive metabolites can be efficiently imported into root cells, as indicated by the upregulation of a large group of genes for plasma membrane amino acid, nucleotide and sugar transporters.

Aerial cells (ECM-free cells) behave like less active cells in synthetic (anabolic) metabolic pathways but our data indicate that they upregulate selected catabolic pathways that can potentially provide them with building blocks for cell reprogramming. The upregulation of genes involved in peroxisomal functions, including β-oxidation of fatty acids, which can provide metabolic intermediates such as acetyl CoA, is a prominent feature of aerial cells. Additional metabolic intermediates may be provided by autophagy, which is active in aerial parts of colonies, as shown by the vacuolar localization of cytosolic and peroxisomal proteins. Aerial cells also express genes for the synthesis and degradation of the storage glucose polymers/ dimers, glycogen and trehalose and accumulate high levels of glycogen, which is usually a sign of nutrient limitation [29]. The activity of glycogen synthase Gsy2p, the major enzyme involved in glycogen synthesis, is regulated by phosphorylation, whereby Ser/Thr protein phosphatase Glc7p dephosphorylates and activates Gsy2p. Glc7p is targeted to Gsy2p by a specific targeting subunit Gac1p [16]. Pig1p, Pig2p and Gip2p, identified as Gsy2p interacting proteins, may be additional Glc7p targeting subunits involved in glycogen synthesis [30, 31], although only deletion of the *PIG2* gene was found to affect glycogen level [32]. In aerial cells of colony biofilms, all *PIG1*, *PIG2* and *GIP2* genes are highly expressed and single deletion of any of these genes decreased glycogen levels. Deletion of the *GIP2* gene decreased glycogen levels in aerial cells to ~19%, arguing for a major role of Gip2p in regulation of glycogen synthesis in colony biofilms. These data, together with previous contradictory findings as to whether deletion of the *PIG2* gene did or did not have an effect on glycogen level and the finding that single deletions of the other two genes *PIG1* and *GIP2* have no effect on glycogen level, imply that alternative Glc7p targeting subunits regulate glycogen synthesis under different growth conditions.

The above-mentioned characteristics are consistent with the conclusion that aerial cells are starved of nutrients. This conclusion is additionally supported by the observed activation of isogenes that are upregulated in liquid culture cells under sugar-limited conditions and is consistent with the finding that significant proportions of aerial cells have entered the stationary-phase in 3-day-old colonies [3]. Further support is provided by the observed expression of genes involved in stress responses, including desiccation stress, oxidative stress and chaperones involved in protein folding. Aerial cells express also a large group of genes involved in meiosis and sporulation, although sporulation is only rarely observed in BR-F colonies, and the BR-F strain does not sporulate efficiently even in liquid medium (unpublished). However, upper cells of both smooth and structured colonies of other diploid *S. cerevisiae* strains grown on sporulation acetate medium exhibit efficient meiosis and sporulation [33, 34].

Aerial and root cells exhibit some similarities to and more differences from U cells (cells in upper regions) and L cells (cells in lower colony regions, which, however, do not invade the agar) of differentiated smooth colonies [9]. The similarities include active autophagy and glycolysis, higher levels of storage compounds (glycogen and trehalose) in U cells and aerial cells than in L and root cells, respectively, and higher expression of genes for gluconeogenesis in both L and root cells than in U and aerial cells, respectively. The numerous differences observed between aerial/U cells and root/L cells clearly reflect the different multicellular lifestyles of colonies and colony biofilms. Slowly dividing U cells with a longevity phenotype activate an unusual metabolism with features typical of metabolically active cells (e.g., activating amino acid and carbohydrate metabolic pathways, accumulating high levels of intracellular amino acids and expressing ribosomal and translation genes) combined with features typical of stationary-phase cells (such as active autophagy and accumulation of storage compounds). In contrast, aerial cells behave mostly like typical stationary-phase cells. Aerial cells express, more highly than root cells, peroxisomal and other catabolic pathway genes that may provide vital compounds to cells under nutrient limitation and numerous stress-related genes that help to make aerial cells more resistant to environmental impact. Other features typical of U cells, such as a decreased expression of genes involved in mitochondrial oxidative phosphorylation, are absent in aerial cells. Regarding the L/root cell comparison, whereas densely packed starving L cells behave like atypical stationary-phase cells (lacking some stationary-phase cell features such as autophagy and accumulation of storage compounds [9]), ECM-embedded root cells behave like metabolically active cells that activate genes for amino acid and nucleotide metabolism and for multiple nutrient transporters, ribosomal genes and genes involved in translation. The ECM plays an important role in these differences because it helps to organize cells within colony biofilms into a network with many small chambers and channels and thus increases the effectiveness of nutrient flow and waste removal through passive capillary movement.

## Conclusions

In summary, our data provided complex information on colony biofilm properties and on processes with important functions in biofilm formation and/or functions of individual cell types. For example, taking together the fact that root cells are embedded within ECM and are highly metabolically active as predicted here, some of their metabolic activities could be expected to contribute to ECM production. Inspiration in this regard could come from bacteria, where activation of gluconeogenesis, one of the features of root cells, was shown to be important for production of exopolysaccharides, a component of ECM in bacterial colony biofilms and further related to bacterial biofilm formation [35]. Glycogen accumulation, typical of aerial cells, was related to bacterial ability to adapt and settle in new niches, including the host organism [36, 37] and could have similar function in yeast cells localized to environment-facing parts of yeast biofilms. In natural conditions, these aerial cells can be easily dispersed and settled into new territories, using glycogen for appropriate metabolic reprogramming. Targeting of Gip2p orthologues in pathogens may then have relevance in antifungal therapy. Further research is needed to verify these hypotheses.

## Methods

### Strains and media

The wild diploid yeast strain BR-F [3, 4] was supplied from the collection of the Chemical Institute of the Academy of Science in Bratislava, Slovak Republic (collection number CCY 21-4-97). All other strains were derived from the BR-F strain and were prepared in this study (Additional file 2: Table S7). Strains with C-terminal GFP fusions were constructed using a GFP-KanMX integrative cassette, amplified by PCR from plasmid pKT127 [38] or pYM27 [39]. Gene deletions were prepared according to [40] and [41]. The primers and plasmids are listed in Additional file 2: Table S8. The yeast cells were transformed using the lithium acetate method as described by [42]. Positive transformants were selected on GMA (3% *v*/v glycerol, 1% yeast extract, 2% agar) supplemented with G418 or nourseothricin (NTC).

Colonies were grown on GMA at 28 °C unless otherwise indicated. GMA supplemented with 200 mg/l G418 (Duchefa Biochemie, The Netherlands) or 100 mg/l NTC was used for selection of the transformants.

### Separation of aerial and root cells from colony biofilms

A polycarbonate track-etched membrane (pore size 5 μm) was placed on the surface of GMA with low agar content (3% glycerol, 1% yeast extract, 0.3% agar) to facilitate separation of the aerial and root parts of the BR-F microcolonies. Cells of the BR-F strain were spread over a membrane on GMA at densities of 1.5 × 10^3^–2 × 10^3^ per plate and microcolonies were grown at 28 °C. Biomass was harvested 72 h after plating. The membrane was removed from the GMA, aerial colony parts were harvested and immediately frozen in liquid nitrogen. Agar medium with embedded root cells of microcolonies was mixed with a spatula, vortexed and the tubes were centrifuged at 20,000 g at 22 °C for 5 min. The resulting pellet (containing root cells together with traces of the agar) was transferred into a new tube and immediately frozen in liquid nitrogen. The aerial and root samples were stored at −80 °C until use.

For each experiment, a large number of plates were inoculated. All biomass was harvested at a precise time point of colony development (72 h after plating) to eliminate possible age-related differences in RNA expression among the parallel samples.

### RNA isolation and DNase treatment

Biomass from BR-F microcolonies (100 mg for the aerial part and 150 mg for the root part containing traces of the agar) was suspended in 400 μl TES buffer (10 mM Tris pH 7.5, 10 mM EDTA, 0.5% SDS). The total RNA was extracted using the hot-phenol extraction procedure as previously described [43]. To verify the quality and quantity of the RNA extracted, spectrophotometric and electrophoretic analyses were performed. The absorbance of the samples were analyzed at 230, 260, and 280 nm. The results of denaturing gel electrophoresis confirmed the presence of high-quality RNA without detectable degradation. The yield of total RNA varied depending on sample type. On average, our protocol obtained 1 mg total RNA from 1 g of aerial sample (composed of pure cell biomass) and 0.1 mg total RNA from 1 g of root sample (composed of root cell biomass and traces of agar medium). Because of traces of agar medium in the root samples, the method does not allow evaluation of the relative RNA amount in roots and aerial cells. The total RNA (20 μg) from each replicate was DNase treated using a GenElute™ Mammalian Total RNA Miniprep Kit (Sigma-Aldrich).

### cDNA library preparation and RNA sequencing

The ribosomal RNA was depleted from all samples using the Ribo-Zero Gold (yeast) rRNA removal kit (Illumina Inc., San Diego, CA) followed by purification using Agencourt RNAClean XP reagents (Beckman Coulter, Brea, CA). A total of 1.15 μg total RNA was used per sample. Efficient rRNA removal was confirmed using a 2100 Bioanalyzer system (Agilent Technologies, Santa Clara, CA). Sequencing libraries were prepared from 50% of the depleted rRNA using a TruSeq Stranded Total RNA Library Prep Kit (Illumina Inc., San Diego, CA) with adapters 2, 4, 5, 6, 7 and 12 for 15 PCR amplification cycles. The libraries were purified using Agencourt AMPure XP beads (Beckman Coulter, Brea, CA) according to the manufacturer’s instructions. A Qubit 3.0 Fluorometer (Thermo Fisher, Waltham, MA) and 4200 TapeStation (Agilent Technologies, Santa Clara, CA) were used to assay the concentration and quality. Paired-end sequencing was conducted on a single lane of an Illumina HiSeq 2500 system (Illumina, San Diego, CA) with 100 bp reads.

### Read mapping

We generated a transcript database by combining the *S. cerevisiae* build R64 coding loci [Ensembl release 76, [44]] with trimmed, de-duplicated non-coding loci from [20–24] to produce a reference GTF file. To avoid duplication, loci from the first three non-coding sources (that had minimal overlap) were given priority over the last two and duplicate loci were deleted accordingly. The lncRNA loci fall into two groups: stable SUTs and unstable CUTs, MUTs and XUTs. The sequencing reads were mapped to the SacCer3 reference using TopHat v2.0.14 [45]. We employed the standard two-step procedure that attempts to map all reads to the transcript database (stored in a GTF file) and then attempts to map unmapped reads to the genomic sequence.

### Differential expression analysis and comparison of samples and replicates

Reads that mapped to genomic features from the reference GTF file were counted in a strand-specific manner using the feature-Counts function from the Rsubread package [46], v1.22.2, of Bioconductor. We then compiled the percentage of reads that mapped to different feature categories: annotated coding genes, mitochondrial coding genes, intergenic regions, dubious ORFs, retrotransposons, and lncRNA i.e. non-coding loci more than 200 bp in length and annotated during five previous studies [20–24]. Finally, differential gene expression analysis was carried out on the read counts using the Bioconductor package DESeq2 v1.12.3 with default settings [47], under the R environment version 3.3.1. DESeq2 results were then exported to Microsoft Excel for further analysis.

### Functional analysis of transcriptomic data

The DESeq differential expression results table was annotated with gene names obtained using the *Saccharomyces cerevisiae* Gene Identifier Converter (http://www.rothsteinlab.com/tools/apps/orf_converter) and with functional grouping based on gene ontology terms obtained using the “Funspec” Functional specification tool (http://funspec.med.utoronto.ca/), supplemented by detailed gene information from the *Saccharomyces cerevisiae* Genome database ([48]; http://www.yeastgenome.org/) and the literature. Unless otherwise stated, genes were regarded as differentially expressed if their *p*-value was below 0.05 and there was a greater than two-fold difference in expression. We also computed *p*-values corrected for multiple testing (both FDR and Bonferroni corrections). Further analysis was conducted in Excel (2013) using appropriate functions. Metabolic network diagrams were constructed based on known functional roles of gene products, differential expression results from this study and pathway information from the Yeast Pathways Database (http://pathway.yeastgenome.org/).

### Secreted proteins, cell wall and adhesins

*S. cerevisiae* orthologues of *Candida albicans* genes encoding secreted proteins [19] were identified with the help of the Candida Genome Database [49]. The list of *S. cerevisiae* orthologs of *C. albicans* secreted protein genes was annotated with differential expression results from this study.

### Northern blotting

Fifteen micrograms of total RNA were denatured in loading buffer with formamide, separated in 1.5% agarose gel and then transferred to a positively charged nylon membrane (Amersham HybondTM-XL, GE Healthcare Ltd). The membranes were hybridized with specific DNA probes prepared using a random primer labelling kit (Takara). The PCR fragments of particular genes (Fig. 3a) were labelled with [α-32P] dCTP. The rRNA content was visualized by ethidium bromide staining and used as a loading control.

### Microscopic analysis of cells within the colony structure

Microcolonies (3-day-old) of GFP-labeled strains were visualized by 2PE-CM according to [50]. In brief, colonies were embedded in low-gelling agarose and cut vertically down the middle. The cut surface was placed on a coverslip, and colony side views were obtained by 2P-CM. A true confocal scanning microscope (SP8 AOBS VLL MP; Leica) was used, fitted with a mode-locked laser (Ti:Sapphire Chameleon Ultra; Coherent Inc.) for two-photon excitation and 20×/0.70 and 63×/1.20 water immersion plan Apochromat objectives. An excitation wavelength of 920 nm was used with emission bandwidths set to 480–595 nm. Images of microcolonies were composed of two stitched fields of view.

### Staining of colony cross-sections for presence of glycogen

Vertical cross sections of 3d old microcolonies were prepared as described previously [9]. In brief, microcolonies were embedded in 2% agarose gel and sectioned using a Leica VT1200S vibrating microtome. Sections from the central colony part were stained by iodine vapor for 3 min and immediately examined by light microscopy. Brown coloration of cells stained by iodine vapor is proportional to their glycogen content [51].

### Quantification of glycogen and trehalose in aerial cells

The total-cell lysate was prepared from 100 to 150 mg of wet aerial cell biomass. The cells were broken with glass beads in 10 mM MES buffer, pH 6, supplemented with Complete, EDTA-free protease inhibitor mixture (Roche Applied Science) and 1 mM AEBSF [4-(2-aminoethyl)benzenesulfonyl fluoride, Sigma] in a FastPrep (Qbiogene). Cell debris was removed by centrifugation for at 1000 g, 3 min, 4 °C and subsequently 3000 g, 5 min, 4 °C. The protein concentration in the resulting cell lysate was determined using a protein detection kit (Bio-Rad) [52]. Glycogen and trehalose in the cell lysate were determined as described in [53]. Briefly, the protein lysate was resuspended in 0.25 M Na_2_CO_3_, and incubated at 95 °C for 4 h. The pH of lysate was then adjusted by addition of 1 M acetic acid and 0.2 M Na-acetate, pH 5.2. One-half of the suspension was incubated overnight with trehalase (0.05 U/ml) at 37 °C, and the second half with amyloglucosidase from *A. niger* (1.2 U/ml) at 57 °C, under constant agitation. The suspensions were centrifuged for 5 min at 5000 g at 4 °C, and glucose was determined by the Glucose (GO) Assay Kit (Sigma-Aldrich).

### HPLC analysis of amino acid content

The aerial and root parts of 3-day-old BR-F microcolonies were separated as described above, with additional washing of root cells with distilled H_2_O, and total intracellular amino acids were extracted from cell suspensions in water by boiling for 5 min. The concentration was determined by HPLC with precolumn derivatization by OPA (o-phthaldialdehyde) [9, 54] with a ZORBAX Eclipse AAA, 3.5 μm, 4.6 × 75 mm reverse phase column (Agilent), and fluorescence detection.

## Additional files

Additional file 1: **Figure S1.** Position clustering of differently expressed genes on chromosomes. **Figure S2.** Intracellular amino acids in aerial and root cells. (PDF 525 kb)

Additional file 2: **Table S1.** Log ratios and *p*-values from DESeq analysis of read counts. **Table S2.** Genes and lncRNAs expressed at a higher level in aerial cells. **Table S3.** Genes and lncRNAs expressed at a higher level in roots. **Table S4.** *S. cerevisiae* orthologs of *C. albicans* secreted proteins. **Table S5.** Mapping and relative expression of lncRNA to lncRNA loci in GTF file. **Table S6.** All lncRNAs upregulated 2-fold in aerial or root and lying within 1.5 kB of a coding gene. **Table S7.** Yeast strains used in this study. **Table S8.** Primers and plasmids used in this study. (XLSX 2047 kb)

## Abbreviations

2PE-CM: Two-photon excitation confocal microscopy
CUTs: Cryptic unstable transcripts
ECM: Extracellular matrix
FDR: False discovery rate
GO: Gene Ontology
lncRNA: long non-coding RNA
MUTs: Meiotic unstable transcripts
PPP: Pentose phosphate pathway
RNA-seq: RNA sequencing
SUTs: Stable unannotated transcripts
TCA: Tricarboxylic acid
XUTs: Xrn1p-dependent unstable transcripts
