# Supplementary material for: Metabolic differentiation of surface and invasive cells of yeast colony biofilms revealed by gene expression profiling
Source: BMC Genomics. 2017 Oct 23;18:814. doi: 10.1186/s12864-017-4214-4 (PMC5654107; doi:10.1186/s12864-017-4214-4)
Supplement: Supplementary file 1 — Figure S1. Position clustering of differently expressed genes on chromosomes. Figure S2. Intracellular amino acids in aerial and root cells. (PDF 525 kb) [file 12864_2017_4214_MOESM1_ESM.pdf]

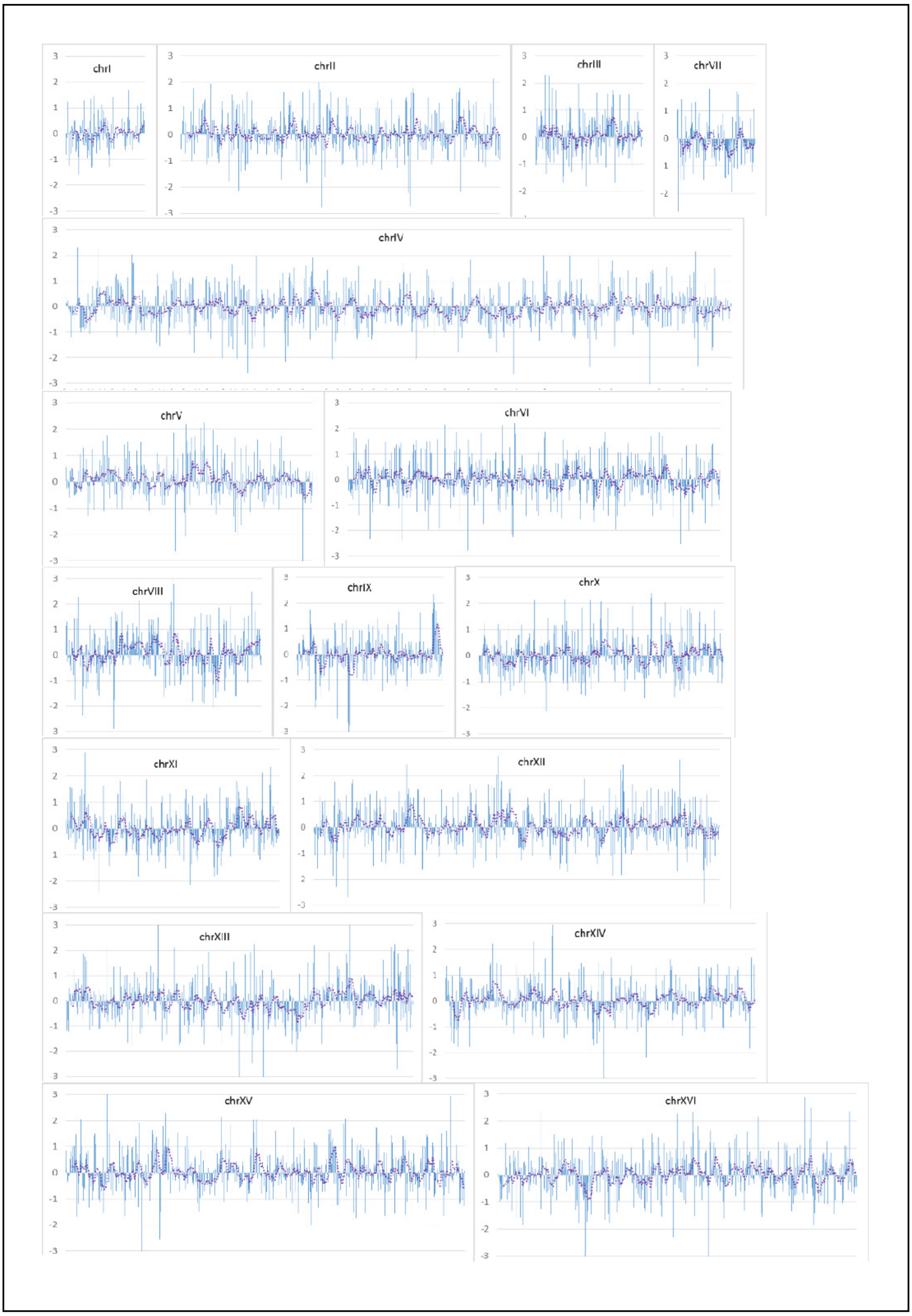

Figure S1: Position clustering of differently expressed genes on chromosomes. For individual chromosomes expression ratios (root/aerial) was plotted against the loci IDs and the moving log average (of 10 loci) was added to the plot as a trend line.

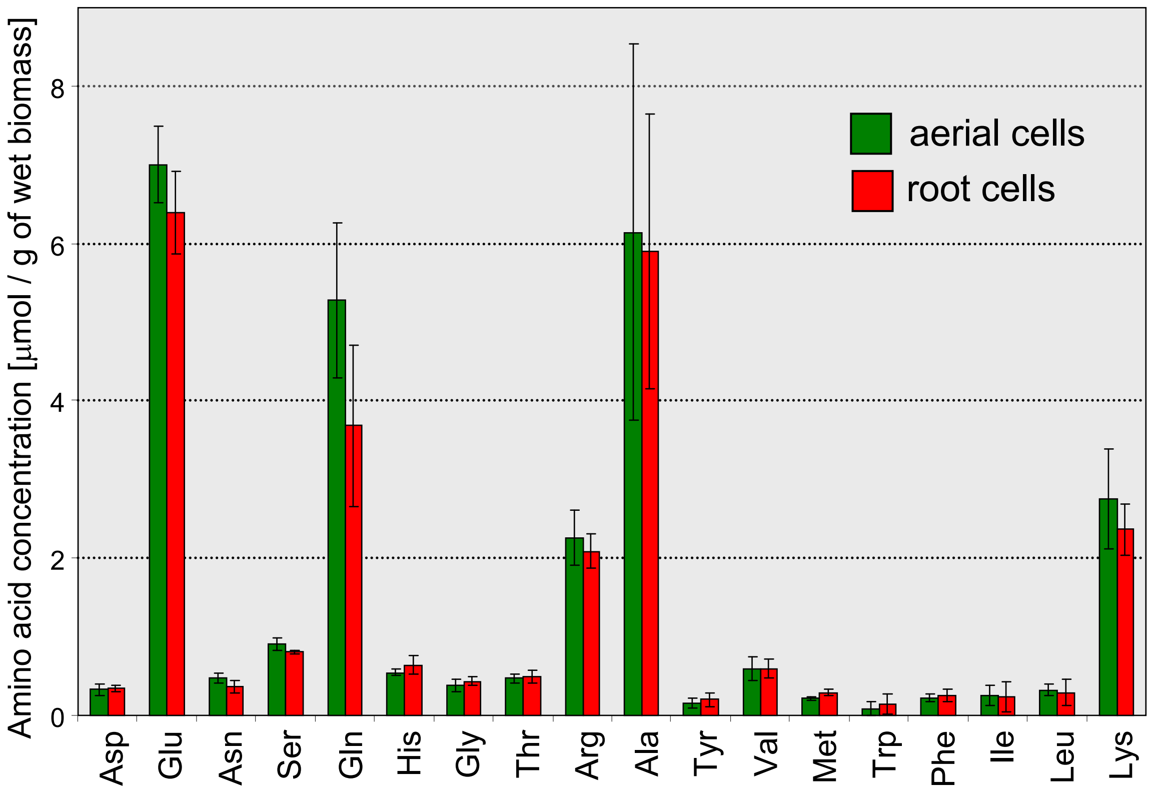

Figure S2: Intracellular amino acids in aerial and root cells. Concentrations of intracellular amino acid were compared in aerial (green) and root (red) cells by HPLC. Average values are shown of 8 independent biological replicates ± S.D.
